# Comparative analysis of serum anti-Müllerian hormone (AMH) levels in sheep: the role of genetic background and physiological status

**DOI:** 10.5194/aab-69-37-2026

**Published:** 2026-01-14

**Authors:** Uğur Kara, Davut Koca

**Affiliations:** 1 Cukurova University, Faculty of Veterinary Medicine, Department of Obstetrics and Gynecology, Adana, Türkiye; 2 Van Yuzuncu Yil University, Faculty of Veterinary Medicine, Department of Obstetrics and Gynecology, Van, Türkiye

## Abstract

Anti-Müllerian hormone (AMH) serves as a reliable marker for ovarian reserve and reproductive potential in many species. However, there is limited information available on the factors influencing AMH levels in sheep. Thus, this study aimed to evaluate the effects of breed, age, body condition score (BCS), and parity on serum AMH concentrations in four commercial sheep breeds: Lacaune, Assaf, Île de France, and Merino. A total of 120 clinically healthy ewes aged between 19 and 34.5 months were included, with equal representation across breeds and lactation stages. AMH levels were measured using standardized laboratory techniques. Significant differences in serum AMH concentrations were observed among breeds ( p<0.001 ). Île de France and Merino breeds exhibited significantly higher mean AMH levels (479.0 and 391.0 $\mathrm{pg}\;{\mathrm{mL}}^{-1}$ respectively) compared to Lacaune and Assaf breeds (261.3 and 205.3 $\mathrm{pg}\;{\mathrm{mL}}^{-1}$ , respectively), suggesting a larger ovarian follicular reserve in these breeds. The range of AMH values was widest in Île de France sheep, indicating considerable intra-breed variability. No statistically significant effects of age, BCS, or parity on serum AMH concentrations were detected ( p>0.05 ). These findings highlight the predominant influence of genetic factors on ovarian reserve in sheep as reflected by serum AMH levels. The elevated AMH concentrations in Île de France and Merino breeds may be linked to superior reproductive potential. Understanding breed-specific variations in AMH could support selective breeding programs aimed at enhancing sheep reproductive efficiency and productivity.

## 1 Introduction

Anti-Müllerian hormone (AMH) is a dimeric glycoprotein hormone that is expressed by granulosa cells of primary, pre-antral, and small antral follicles. Its expression progressively decreases in the subsequent stages of follicular development, leading to the cessation of AMH expression during the gonadotropin-dependent stages of follicle development. Additionally, AMH expression ceases when follicles undergo atresia (Durlinger et al., 2002; Themmen, 2005). AMH plays a critical inhibitory role in modulating the responsiveness of both granulosa and theca cells to gonadotropic stimuli, thereby regulating the pace at which follicles advance from the gonadotropin-responsive stage to the gonadotropin-dependent stage (Campbell et al., 2012).

Research on AMH and ovarian reserve spans various species, with a notable focus on women and different animal species. In women, AMH is related to the number and quality of oocytes (Heidary et al., 2024). In addition, its serum levels are used for the diagnosis of diseases such as polycystic ovary syndrome (van der Ham et al., 2024) and premature ovarian insufficiency (Khudhair et al., 2024), as well as its implications in clinical outcomes of patients with in vitro fertilization–intracytoplasmic sperm injection (IVF-ICSI) (Elmoutabi et al., 2024), and AMH cut-off levels are used to predict post-treatment ovarian function in patients with early breast cancer (Omranipour et al., 2024), pregnancy outcomes, and spontaneous abortion (Liang et al., 2024; Yilei et al., 2024). Recent studies in bitches have focused on the half-life of AMH (Balogh et al., 2024), its relationship with litter size (Hornberger et al., 2025), its usefulness in determining the stage of the estrous cycle (de Oliveira et al., 2024), the monitoring of AMH levels in pregnant and non-pregnant females (Evci et al., 2023), and its association with endometrial polyps (Lai et al., 2023). Studies in queens have investigated AMH levels in various body fluids (Kaya et al., 2024), the expression of AMH and its receptor AMHRII (Gültiken et al., 2022), factors influencing AMH concentrations (Gözer et al., 2024), and AMH levels during the follicular and interestrus period (Piryagci et al., 2024). In contrast, research on AMH and ovarian reserve in sheep remains relatively limited.

AMH is commonly acknowledged as the most reliable hormonal marker for evaluating ovarian reserves; however, there is evidence indicating that its expression and serum levels can be influenced by a variety of factors (Bhide et al., 2015; Gleicher et al., 2009; Schuh-Huerta et al., 2012; Tal and Seifer, 2013). Factors influencing AMH levels in women include race, age, smoking habits, and vitamin D deficiency (Shahrokhi et al., 2018; Tal and Seifer, 2013). Studies have reported that factors influencing AMH levels in ruminants include puberty, fetal sex, diseases, genetics, and heat stress (Alward and Bohlen, 2020; Mossa et al., 2017; Umer et al., 2019). Breed has a significant effect on AMH levels in cattle. Studies generally show that meat-type breeds have higher AMH concentrations than dairy breeds (Mossa et al., 2017). For example, Charolais, Angus, Nelore, and Gyr heifers exhibit higher AMH levels than Holstein, Jersey, and Murrah breeds (Pfeiffer et al., 2014; Batista et al., 2014; Baldrighi et al., 2014). However, some variation exists among dairy breeds, with Jersey cows showing relatively higher AMH concentrations than Holstein cows (Ribeiro et al., 2014).

The sheep industry is a vital branch of animal husbandry, characterized by high productivity and a diverse range of products, including wool, meat, leather, and milk (Joy et al., 2020; Ralph Clark et al., 2021). Although studies on AMH in sheep are limited, recent research is of great importance in terms of reproduction and genetic progress (Turgut and Koca, 2024a, b). Studies in cattle suggest that AMH may also be affected by breed in sheep. On the other hand, litter size may differ by breed in sheep. It is thought that this difference may be due to the ovarian reserve of the ewes. A novel study revealed that litter size may be related to serum AMH, possibly due to high ovarian reserve in Romanov sheep (Turgut and Koca, 2024b). Regarding the importance of higher litter size in sheep for the sustainability of animal production, the detection and comparison of serum AMH levels in different sheep breeds may be valuable for further studies that focus on production traits. According to current knowledge, this study is among the first to investigate the effect of breed on AMH levels in four different sheep breeds, including two meat-type and two dairy-type breeds.

## 2 Material and methods

### 2.1 Animals

A total of 120 clinically healthy ewes, aged between 19 and 34.5 months, were used in the study, with 30 animals from each of the following breeds: Lacaune, Assaf, Île De France, and Merino. The ewes included in this study were maintained under similar environmental conditions. The ewes were housed under farm conditions that included indoor shelters and free-access outdoor areas. Their diet consisted of a legume–cereal hay mixture, straw, corn silage, a commercial concentrate feed (containing 19 % crude protein, 3.60 % fat, and 2700 $\mathrm{kcal}\;{\mathrm{ME}}^{-1}$ ), and barley. Water and mineral blocks were provided ad libitum. For the Île De France and Merino breeds, only non-lactating ewes that had weaned their lambs at least 30 d before the study were selected. Assaf and Lacaune ewes were milked twice daily using a 2×12 fishbone milking system. Body condition score (BCS) ranged between 3.0–3.5 for the Lacaune breed and 3.5–4.0 for the other breeds (on a 1–5 scale). The ewes were periodically vaccinated against sheep pox, enterotoxemia, mastitis, and pasteurella according to the vaccination program. Internal and external parasite medications were administered twice a year.

### 2.2 Collection of blood samples

All blood samples were collected once in March 2025. Blood samples were collected between 10:00 and 12:00 (a.m.) via jugular venipuncture. Blood samples were taken from the jugular vein using a 0.8×38mm (21 G) cannula through 9 mL tubes containing clot activator (AYSET, Türkiye). In this study, AMH levels were evaluated in sheep. Recent research indicates that both in-season and off-season AMH concentrations are not affected by the different stages of the estrous cycle and remain relatively stable, suggesting that a single measurement can reliably reflect circulating AMH levels. Consequently, numerous studies have been conducted based on this premise (Brochado et al., 2024; Turgut and Koca, 2024a, b; Lahoz et al., 2014; Çetin and Koca, 2025). The samples were centrifuged at 2000×g for 15 min, and the serum was stored at -80°C until analysis for AMH determination.

### 2.3 AMH assay

Samples were analyzed to determine AMH concentrations using the electrochemiluminescence immunoassay (ECLIA) method, as previously described (Koca et al., 2023, 2024b; Turgut and Koca, 2024a, b; Çetin and Koca, 2025). AMH measurements were performed with the Elecsys^®^ AMH automated assay on the Cobas 601 platform (Roche, Germany). Prior to the main analysis, the assay method was validated in accordance with the manufacturer's instructions. Calibration and standard curves were evaluated based on accurately assigned reference values. Furthermore, the Elecsys^®^ AMH assay has a validated measuring range of 0.01–23 $\mathrm{ng}\;{\mathrm{mL}}^{-1}$ .

### 2.4 Statistical analysis

Minitab (v21.4.1) was used for the statistical analysis of the data. Due to the normal distribution of the data, parametric tests were preferred for analysis. A one-way ANOVA test was used to determine the effect of breed on AMH. Pearson correlation analysis was performed to evaluate the relationship between age (months) and AMH levels. An independent-sample t test was used to evaluate the impact of lactation and BCS on serum AMH. The distribution of AMH data in each breed was visualized using a boxplot. A value of p<0.05 was considered to be at the statistically significant level.

## 3 Results

The serum AMH concentrations varied notably among the four sheep breeds studied. The Île de France breed exhibited the broadest range of AMH values, covering from 100.0 $\mathrm{pg}\;{\mathrm{mL}}^{-1}$ (minimum) to 1080.0 $\mathrm{pg}\;{\mathrm{mL}}^{-1}$ (maximum), indicating considerable variability in ovarian reserve within this breed. Similarly, the Merino breed showed a broad range between 110.0 and 940.0 $\mathrm{pg}\;{\mathrm{mL}}^{-1}$ . In contrast, the Lacaune breed's AMH values ranged from 90.0 to 540.0 $\mathrm{pg}\;{\mathrm{mL}}^{-1}$ , while the Assaf breed presented the narrowest range, between 70.0 and 410.0 $\mathrm{pg}\;{\mathrm{mL}}^{-1}$ (Table 1).

**Table 1:** Descriptive statistics of serum AMH levels in Lacaune, Assaf, Île de France, and Merino sheep breeds.

Variable	N	Mean	SE	SD	Minimum	Q1	Median	Q3	Maximum
Lacaune	30	261.3	21.6	118.2	90.0	180.0	225.0	340.0	540.0
Assaf	30	205.3	14.1	77.4	70.0	157.5	205.0	240.0	410.0
Île de France	30	479.0	45.2	24.6	100.0	265.0	440.0	645.0	1080.0
Merino	30	391.0	39.7	217.7	110.0	200.0	330.0	602.5	940.0

SE: Standard error of mean. SD: Standard deviation

Considering BCS, in the Lacaune breed, mean serum AMH concentrations were 265.6 and 256.4 $\mathrm{pg}\;{\mathrm{mL}}^{-1}$ in the BCS 3.0 and 3.5 groups, respectively, with no statistically significant difference between the groups ( p>0.05 ). Standard deviation values were similar, indicating consistent inter-individual variability. In the Assaf breed, mean AMH levels were 206.4 $\mathrm{pg}\;{\mathrm{mL}}^{-1}$ in the BCS 3.5 group and 204.7 $\mathrm{pg}\;{\mathrm{mL}}^{-1}$ in the BCS 4.0 group. No statistically significant difference was observed between these groups ( p>0.05 ). In the Île de France breed, serum AMH concentrations were 474.4 $\mathrm{pg}\;{\mathrm{mL}}^{-1}$ for the BCS 3.5 group and 484.3 $\mathrm{ng}\;{\mathrm{mL}}^{-1}$ for the BCS 4.0 group. This difference was not statistically significant ( p>0.05 ). In the Merino breed, mean AMH levels were 398.9 and 377.3 $\mathrm{pg}\;{\mathrm{mL}}^{-1}$ in the BCS 3.5 and 4.0 groups, respectively, with no significant difference between them ( p>0.05 ) (Table 2).

**Table 2:** The effects of BCS on serum AMH in sheep.

Breed	BCS	N	Mean	SD	p value
Lacaune	3.0	16	265.6	116.2	0.836^NS^
	3.5	14	256.4	124.8	
Assaf	3.5	11	206.4	82.1	0.957^NS^
	4.0	19	204.7	76.8	
Île de France	3.5	16	474.4	244.9	0.91^NS^
	4.0	14	484.3	259.9	
Merino	3.5	19	398.9	224.9	0.798^NS^
	4.0	11	377.3	214.5	

^NS^ Non-significant.

**Figure 1:**
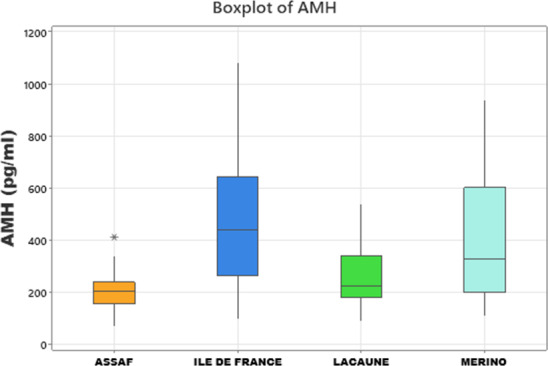
Boxplots showing serum AMH levels in Assaf, Île de France, Lacaune, and Merino sheep breeds.

**Figure 2:**
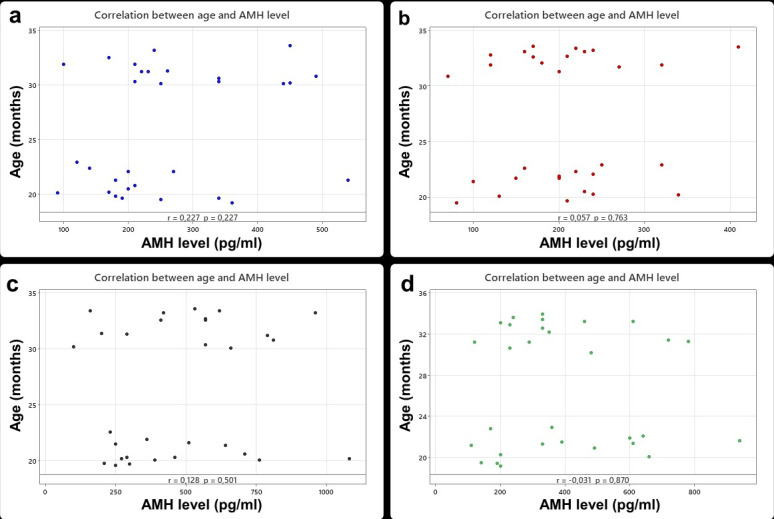
Correlation between age and AMH level in Lacaune **(a)**, Assaf **(b)**, Île de France **(c)**, and Merino **(d)** breeds.

In the sheep breeds, mean serum AMH levels did not change between primiparous ewes and multiparous ewes ( p>0.05 ) (Table 3). However, the results revealed a highly significant effect of breed on serum AMH concentrations ( p<0.001 ). Specifically, Île de France (479.0 $\mathrm{pg}\;{\mathrm{mL}}^{-1}$ ) and Merino (391.0 $\mathrm{pg}\;{\mathrm{mL}}^{-1}$ ) breeds exhibited significantly higher mean AMH levels compared to Lacaune (261.3 $\mathrm{pg}\;{\mathrm{mL}}^{-1}$ ) and Assaf (205.3 $\mathrm{pg}\;{\mathrm{mL}}^{-1}$ ) breeds. AMH concentrations in Île de France and Merino are significantly greater than those in Lacaune and Assaf (Fig. 1). However, the differences between Assaf and Lacaune were not significant. Similarly, differences between Merino and Île de France were also insignificant ( p>0.05 ) (Table 4). Also, there was no correlation between age and serum AMH level in Lacaune ( r=0.027 ), Assaf ( r=0.057 ), Île de France ( r=0.128 ), and Merino breeds ( r=-0.031 ) ( p>0.05 ) (Fig. 2).

**Table 3:** The effects of parity on serum AMH in sheep.

Breed	Parity	N	Mean	SD	p value
Lacaune	1	15	229.3	113.2	0.141^NS^
	2	15	293.3	118.1	
Assaf	1	15	204.7	72.8	0.963^NS^
	2	15	206.0	84.2	
Île de France	1	15	447.3	250.5	0.493^NS^
	2	15	510.7	249.3	
Merino	1	15	402.0	245.6	0.787^NS^
	2	15	380.0	193.8	

^NS^ Non-significant.

**Table 4:** Comparison of serum AMH levels between sheep breeds.

Breed	N	Mean	SD	p value
Lacaune	30	261.3^b^	118.2	0.000*
Assaf	30	205.3^b^	77.4	
Île de France	30	479.0^a^	247.6	
Merino	30	391.0^a^	217.7	

* p<0.001 , ^a,b^ Different letters show significant differences between groups.

## 4 Discussion

AMH is a glycoprotein belonging to the transforming growth factor-beta (TGF- β ) family and plays a key role in various biological processes (Papas et al., 2021). In sheep and other animal species, AMH serves as an essential clinical and phenotypic marker for assessing ovarian follicular reserve and superovulatory response (Monti, 2025). Studies have demonstrated that AMH levels vary significantly across breeds, influenced by factors such as genotype and genetic mutations (Xu et al., 2025). Additionally, AMH concentrations in ruminants are influenced by various factors, including puberty, fetal sex, disease status, genetic background, and heat stress (Alward and Bohlen, 2020; Mossa et al., 2017; Umer et al., 2019). In sheep, AMH levels are known to be unstable during the prepubertal period but tend to stabilize after puberty, with notable individual variability (Lahoz et al., 2014; Nabenishi et al., 2017; Mossa et al., 2017). Although limited information is available in the literature regarding the factors influencing AMH levels in sheep (Waheeb, 2017), this study provides additional insights into breed-related differences by evaluating the effect of breed on AMH levels in four sheep breeds, including two meat-type and two dairy-type breeds. This investigation considers variables such as age, BCS, and parity, comparing both meat-type and dairy sheep breeds.

AMH is a critical biomarker for assessing reproductive function and has been extensively studied to evaluate age-related changes in both humans and animals. In a study by Tekce and Korkmaz (2021), AMH levels were examined in 6–12-month-old Simmental heifers, revealing a decrease during the prepubertal period, followed by stabilization in later stages. Similarly, a study conducted by Lahoz et al. (2014) on sheep of the Rasa Aragonesa breed revealed considerable variability in AMH levels during the prepubertal phase. However, in the adult phase, AMH levels did not show significant age-related differences. In a study conducted by Acharya et al. (2020), no significant differences were observed in the AMH levels of Katahdin sheep between the ages of 1 and 7 years. Similarly, Turgut and Koca (2024a) reported that age did not significantly affect AMH levels in Romanov sheep, and it did not have any discernible impact on AMH levels in crossbred Hamdani sheep (Turgut and Koca, 2024b). Furthermore, a study on two indigenous sheep breeds in Slovenia found no significant differences in AMH levels when the sheep were categorized into three age groups (Šterbenc et al., 2025). In another study by Çetin and Koca (2025), AMH levels measured across different sexual stages were found to be unaffected in Norduz ewes aged 1–3 years. Consistently with these previous findings, our study, which measured serum AMH levels in meat and dairy ewes aged between 19 and 34.5 months, also found that age did not significantly influence AMH levels. Similarly, there were no significant correlations between age and serum AMH level in Lacaune, Assaf, Île de France, and Merino breeds.

BCS is a practical visual assessment used to evaluate the body condition of ruminants. It plays a crucial role in nutritional management, monitoring overall health, and assessing both reproductive performance and yield (Blanchard et al., 2025). In a study conducted on Holstein heifers to explore the relationship between BCS and AMH concentrations, animals were categorized into three subgroups based on their BCS: 3.2±0.007 , 3.5±0.006 , and 3.8±0.008 . The corresponding AMH concentrations were measured at 394.9±17.4 , 374.9±16.4 , and $395.5\pm 19.7\;\mathrm{pg}\;{\mathrm{mL}}^{-1}$ , respectively. The results indicated that there was no significant positive correlation between AMH levels and BCS (Machado et al., 2025). In a study conducted on Holstein dairy cows, BCS was found to have no significant effect on AMH levels in individuals with a BCS ranging from 3.0 to 3.5 (Koca et al., 2024a). Similarly, a study on Hamdani crossbred ewes revealed that BCS had no significant impact on AMH levels in individuals with a BCS between 2.75 and 3.25, regardless of whether the animals were in estrus or diestrus (Turgut and Koca, 2024b). Moreover, research on Norduz ewes during the non-reproductive season showed no statistically significant variation in AMH levels in ewes with a BCS between 3.0 and 4.5 (Çetin and Koca, 2025). When considered together with the findings of the present study, these results further support the conclusion that BCS does not significantly influence AMH concentrations in both meat-type and dairy-type sheep breeds.

Numerous studies have investigated the relationship between parity and AMH concentrations, particularly in large ruminants. Koizumi and Kadokawa (2017), for example, reported that, in Japanese Black cows, AMH concentrations were lower in cows that had calved for the first time compared to those with multiple calvings. Similarly, Akbarinejad et al. (2018) found that multiparous Holstein cows exhibited higher AMH levels than nulliparous and primiparous cows. In another study on Holstein cows (Gobikrushanth et al., 2019), it was observed that serum AMH levels varied with parity, with significantly higher plasma AMH concentrations in second-parity cows compared to those in their first parity. In contrast to the relatively extensive research in cattle, studies examining the relationship between parity and AMH levels in sheep are limited. Therefore, the present study also evaluated this association in ewes. However, no significant relationship was found between parity and AMH concentrations in the current investigation. These findings highlight the need for further research to better understand the association between parity and AMH levels in sheep.

Several studies have investigated the influence of breed on AMH concentrations in sheep. In particular, two indigenous Slovenian breeds, Jezersko-Solčava (JS) and Istrska Pramenka (IP), were studied. The results indicated that serum AMH levels were significantly higher in JS sheep compared to in IP sheep. Specifically, AMH concentrations in JS sheep ranged from 1.30 to 21.55 $\mathrm{ng}\;{\mathrm{mL}}^{-1}$ , with a mean value of $4.80\pm 0.63\;\mathrm{ng}\;{\mathrm{mL}}^{-1}$ . In contrast, AMH levels in IP sheep ranged from 0.16 to 4.61 $\mathrm{ng}\;{\mathrm{mL}}^{-1}$ , with a mean concentration of 1.82±0.19 $\mathrm{ng}\;{\mathrm{mL}}^{-1}$ (Šterbenc et al., 2025). Additionally, a study on Norduz sheep reported an average serum AMH concentration of 205 $\mathrm{pg}\;{\mathrm{mL}}^{-1}$ (Çetin and Koca, 2025), while, in Romanov sheep, AMH levels ranged from 40 to 970 $\mathrm{pg}\;{\mathrm{mL}}^{-1}$ , with a mean value of 329 $\mathrm{pg}\;{\mathrm{mL}}^{-1}$ (Turgut and Koca, 2024a). In a study conducted on crossbred Hamdani sheep, serum AMH levels were found to range from 95 to 520 $\mathrm{pg}\;{\mathrm{mL}}^{-1}$ , with a mean value of 247 $\mathrm{pg}\;{\mathrm{mL}}^{-1}$ (Turgut and Koca, 2024b). The present study aimed to compare the serum AMH levels of four different sheep breeds: Lacaune, Assaf, Île-de-France, and Merino. In the Lacaune breed, serum AMH levels ranged from 90.0 to 540.0 $\mathrm{pg}\;{\mathrm{mL}}^{-1}$ , with a mean value of 225.0 $\mathrm{pg}\;{\mathrm{mL}}^{-1}$ . For the Assaf breed, AMH levels varied between 70.0 and 410.0 $\mathrm{pg}\;{\mathrm{mL}}^{-1}$ , and the mean AMH level was 205.0 $\mathrm{pg}\;{\mathrm{mL}}^{-1}$ . In the Île de France breed, serum AMH levels ranged from 100.0 to 1080.0 $\mathrm{pg}\;{\mathrm{mL}}^{-1}$ , with a mean value of 440.0 $\mathrm{pg}\;{\mathrm{mL}}^{-1}$ . In the Merino breed, AMH levels ranged from 110.0 to 940.0 $\mathrm{pg}\;{\mathrm{mL}}^{-1}$ , with a mean of 330.0 $\mathrm{pg}\;{\mathrm{mL}}^{-1}$ . These findings indicate notable differences in AMH levels across sheep breeds. Moreover, this study represents significantly higher AMH levels in meat-type breeds compared to in dairy-type sheep breeds. Both previous data and the results of the current study corroborate the fact that sheep breed significantly influences serum AMH levels, with apparent variations observed between different breeds.

## 5 Conclusions

This study demonstrates that sheep breed is a significant determinant of serum AMH concentrations, reflecting differences in ovarian reserve among breeds. The notably higher AMH levels observed in Île de France and Merino breeds compared to in Lacaune and Assaf suggest a potentially greater reproductive capacity in these meat-type breeds. Conversely, age, BCS, and parity showed no significant influence on serum AMH levels in the studied population, highlighting the predominant role of genetic background over physiological or management factors in shaping ovarian reserve. Furthermore, lactation may be associated with reduced ovarian activity and limited energy availability, both of which can contribute to lower AMH levels. These results highlight the importance of considering lactation status when interpreting AMH concentrations in clinical or research settings. Thus, these findings provide valuable insights for genetic variation and selection programs, emphasizing the importance of considering breed-specific ovarian traits when aiming to improve reproductive efficiency. Further research is warranted to explore the genetic and environmental mechanisms underlying AMH variability and its practical implications for sheep fertility management.

## Data Availability

Any data from our research will be shared upon reasonable request to the corresponding author.
